# Effects of geodemographic profiles on healthcare service utilization: a case study on cardiac care in Ontario, Canada

**DOI:** 10.1186/1472-6963-13-239

**Published:** 2013-07-01

**Authors:** Li Tao, Jiming Liu, Bo Xiao

**Affiliations:** 1 Department of Computer Science, Hong Kong Baptist University, Kowloon Tong, Hong Kong; 2 Information Technology Management Department, Shidler College of Business, University of Hawaii at Manoa, Honolulu, United States of America

## Abstract

**Background:**

Although literature has associated geodemographic factors with healthcare service utilization, little is known about how these factors — such as population size, age profile, service accessibility, and educational profile — interact to influence service utilization. This study fills this gap in the literature by examining both the direct and the moderating effects of geodemographic profiles on the utilization of cardiac surgery services.

**Methods:**

We aggregated secondary data obtained from Statistics Canada and Cardiac Care Network of Ontario to derive the geodemographic profiles of Ontario and the corresponding cardiac surgery service utilization in the years between 2004 and 2007. We conducted a two-step test using Partial Least Squares-based structural equation modeling to investigate the relationships between geodemographic profiles and healthcare service utilization.

**Results:**

*Population size* and *age profile* have direct positive effects on *service utilization* (*β*=0.737, *p*<0.01; *β*=0.284, *p*<0.01, respectively), whereas *service accessibility* is negatively associated with *service utilization* (*β*=−0.210, *p*<0.01). *Service accessibility* decreases the effect of *population size* on *service utilization* (*β*=−0.606, *p*<0.01), and *educational profile* weakens the effects of *population size* and *age profile* on *service utilization* (*β*=−0.595, *p*<0.01; *β*=−0.286, *p*<0.01, respectively).

**Conclusions:**

In this study, we found that (1) *service accessibility* has a moderating effect on the relationship between *population size* and *service utilization*, and (2) *educational profile* has moderating effects on both the relationship between *population size* and *service utilization*, and the relationship between *age profile* and *service utilization*. Our findings suggest that reducing regional disparities in healthcare service utilization should take into account the interaction of geodemographic factors such as service accessibility and education. In addition, the allocation of resources for a particular healthcare service in one area should consider the geographic distribution of the same services in neighboring areas, as patients may be willing to utilize these services in areas not far from where they reside.

## Background

Geodemographic factors, such as population size [1], age profile [2,3], geographic accessibility to services [4], and educational profile [5,6], have been recognized as important determinants of healthcare service utilization [7,8]. Geodemographic factors have conventionally been used to estimate healthcare needs (e.g., population-needs-based funding formula [9]) to improve resource allocation and shorten wait time. The majority of previous work has focused on examining pair-wise relationships between geodemographic factors and healthcare service utilization, with a scarcity of research exploring how the demographic factors interact to affect healthcare service utilization.

Nevertheless, as previous studies have suggested [4,10,11], certain geodemographic factors may moderate (i.e., change the direction and/or strength of) [12] the effects that other geodemographic factors have on healthcare service utilization. For instance, if one area has more healthcare service providers, the burden of population growth and aging on patient arrivals for a specific hospital in that area may be alleviated, as patients residing there have more choices and thus will be more likely to be distributed among multiple hospitals. This suggests that geographic accessibility to services (referred to hereafter as service accessibility) [4] may have potential moderating effects on the relationships between population size/age profile and service utilization. As an additional example, individuals, including seniors, with different educational backgrounds may have varying lifestyles [10] that can influence their risk for cardiovascular disease [5,6] and their healthcare service utilization behavior [11]. This indicates that educational profile may have a potential moderating effect on the relationship between population size and healthcare service utilization.

To the best of our knowledge, no previous studies have explored the potential effects of geodemographic factors such as service accessibility and educational profile in moderating the influence of other geodemographic factors such as population size and age profile on healthcare service utilization. To fill this gap in the literature, we aim to examine both the direct and the moderating effects of geodemographic profiles on cardiac surgery service utilization in various sub-regions of Ontario, Canada. The sub-regions of concern are Local Health Integration Networks (LHINs) [13] in Ontario, Canada. Each LHIN is a geographic-location-based, sub-provincial administrative unit responsible for determining the healthcare service needs and priorities for its corresponding area [13]. In Ontario, there are in total 14 LHINs that differ in their administrative areas, geographic sizes, and geodemographic profiles (as shown in Table 1). While LHINs have been in operation for years, there is a scarcity of academic research examining how geodemographic profiles pertaining to an LHIN influence its healthcare service utilization.

**Table 1:** **The name, size, and scope of LHINs in Ontario, Canada [**[13]**]**

**LHIN ID**	**LHIN name**	**Area**	**PD**	**Boundary**
		**(*km***^***2***^**)**	(**per*km***^***2***^)	**(Major cities/towns/counties)**
1	Erie St. Clair	7323.7	86.1	Windsor, Lambton, Chatham-Kent, and Essex
2	South West	20903.5	43.1	London, Stratford, Elgin, Middlesex, Oxford, Perth, Huron, Bruce, and part of Grey
3	Waterloo Wellington	4746.6	144.6	Wellington, Waterloo, Guelph, and part of Grey
4	Hamilton Niagara Haldimand Brant	6473.0	203.3	Hamilton, Niagara, Haldimand, Brant, and parts of Halton and Norfolk
5	Central West	2590.0	285.7	Dufferin, parts of Peel, York, and Toronto
6	Mississauga Halton	1053.7	956.7	Mississauga, parts of Toronto, Peel, and Halton
7	Toronto Central	192.0	5678.9	A large part of Toronto
8	Central	2730.5	561.3	Parts of Toronto, York, and Simcoe
9	Central East	15274.1	93.8	Durham, Kawartha Lakes, Haliburton Highlands, Heterborough, parts of Northumberland, and Toronto
10	South East	17887.2	26.1	Kingston, Hastings, Lennox and Addington, Prince Edward, and Frontenac
11	Champlain	1763.1	65.1	Ottawa, Renfrew, Prescott and Russell, Stormont, and Dundas and Glengarry
12	North Simcoe Muskoka	8372.3	50.5	Muskoka, parts of Simcoe and Grey
13	North East	395576.7	1.4	Nipissing, Parry Sound, Sudbury, Algoma, Cochrane, and part of Kenora
14	North West	406819.6	0.6	Thunder Bay, Rainy River, and most of Kenora

PD: population density.

To achieve our objective, i.e., to examine the direct and the moderating effects of geodemographic profiles, we construct a conceptual model and develop related hypotheses based on a thorough review of literature. We then test the model with publicly available secondary data representing pertinent geodemographic factors and cardiac surgery service utilization during the four-year period from 2004 to 2007. The data analysis method employed is structural equation modeling (SEM), a second-generation statistical tool efficient in modeling latent variables (those that cannot be directly measured) and testing the complex relationships among the variables [12,14].

### Literature review and research hypotheses

In this study, we explore how geodemographic factors interact to influence healthcare service utilization in the context of cardiac surgery services. These geodemographic factors include population size, age profile, service accessibility, and educational profile. In this section, we review extant literature and develop hypotheses related to the effects of geodemographic profiles (as direct antecedents and moderators) on healthcare service utilization.

#### Population size and service utilization

*Population size*, representing the total population that may utilize the cardiac surgery services in an LHIN, has been shown to exert a direct positive influence on *service utilization* (operationalized as the number of patient arrivals) [1]. A larger population may translate into a greater number of people using healthcare services to prevent or treat various types of illnesses [1, p.59]. Population growth, which may produce more cardiovascular patients, has been identified as one of the major driving forces behind changes in the number of patient arrivals [15]. We thus hypothesize:

**Hypothesis 1 (H1):*Population size* has a direct positive effect on*service utilization***.

#### Age profile and service utilization

*Age profile*, conceptualized as the proportion of seniors (i.e., individuals older than 50) in the population that may utilize cardiac surgery services in an LHIN, has been recognized as another important factor that may influence service utilization. Old age is a traditional cardiovascular risk factor [16]. Other risk factors for cardiovascular disease, such as hypertension, obesity, and physical inactivity, have also been found to be more prevalent in the segment of the population aged 50 and above [17,18]. Further, age groups vary in their healthcare service utilization behavior [2,3], with seniors typically exhibiting a higher rate of utilization. Accordingly, a larger senior population potentially brings in more cardiovascular patients [19], hence leading to a greater number of patient arrivals for healthcare services such as cardiac surgery [15]. Therefore, we hypothesize that:

**Hypothesis 2 (H2):*Age profile* has a direct positive effect on*service utilization***.

#### Service accessibility, population size, age profile, and service utilization

Geographic accessibility to healthcare services in an area (i.e., *service accessibility*) is an important factor influencing patients’ decisions regarding the usage of such services [4,20,21]. Seidel et al. [20] found that a patient’s willingness to utilize healthcare services was negatively associated with the distance between his/her residence and the destination hospital. A survey conducted by Cardiac Care Network (CCN) of Ontario [21] also showed that the driving distance from home to a hospital was one of the most important factors for patients in choosing a specific hospital, and that above 80% of cardiovascular patients were not willing to visit hospitals far away from home. Extending these findings, we conjecture that if there are several accessible hospitals in one area, patient arrivals for any one particular hospital may decrease, as the difference in the time needed for patients to travel to one hospital versus to another is negligible. Under such circumstances, we would expect patients to be dispersed among several hospitals, resulting in reduced wait time for any one particular hospital in this area.

In the context of our study, higher service accessibility for an LHIN implies that residents in that LHIN have access to more alternative healthcare service providers. As a result, the number of patient arrivals for any one particular hospital in the LHIN may decrease. Further, in an LHIN with higher service accessibility, the pressure of population size or age profile on each of the hospitals in the LHIN may be mitigated because patients (including seniors) in the LHIN are likely to be dispersed among several hospitals. Thus we hypothesize:

**Hypothesis 3.1 (H3.1):*Service accessibility* has a direct negative effect on*service utilization***.

**Hypothesis 3.2 (H3.2):*Service accessibility* has a negative moderating effect on the relationship between*population size* and*service utilization***.

**Hypothesis 3.3 (H3.3):*Service accessibility* has a negative moderating effect on the relationship between*age profile* and*service utilization***.

#### Educational profile, population size, age profile, and service utilization

*Educational profile*, defined as the proportion of well-educated individuals (i.e., those with above-high-school education) in the population that may utilize the cardiac surgery services in an LHIN, is an important factor that may also affect healthcare service utilization. Individuals with varied educational backgrounds manifest different lifestyles [10], and are thus associated with varying levels of risk for cardiovascular disease [5,6] and service utilization behavior [11]. For instance, a longitudinal secondary data study in Canada showed that smoking and inactivity, two traditional cardiovascular risk factors, were more prevalent in the less well-educated (senior) population [10]. This study suggested that people in the less well-educated group might have a higher demand for healthcare services related to cardiovascular disease. Another study showed that diabetic patients who were at greater risk for cardiovascular disease were more willing to perform self-care behavior if they were well-educated [11]. These findings suggest that, in addition to directly affecting service utilization, a higher proportion of well-educated individuals in the population may mitigate the pressure of population size and aging on service utilization. Thus, we hypothesize:

**Hypothesis 4.1 (H4.1):*Educational profile* has a direct negative effect on*service utilization***.

**Hypothesis 4.2 (H4.2):*Educational profile* has a negative moderating effect on the relationship between*population size* and*service utilization***.

**Hypothesis 4.3 (H4.3):*Educational profile* has a negative moderating effect on the relationship between*age profile* and*service utilization***.

**Figure 1:**
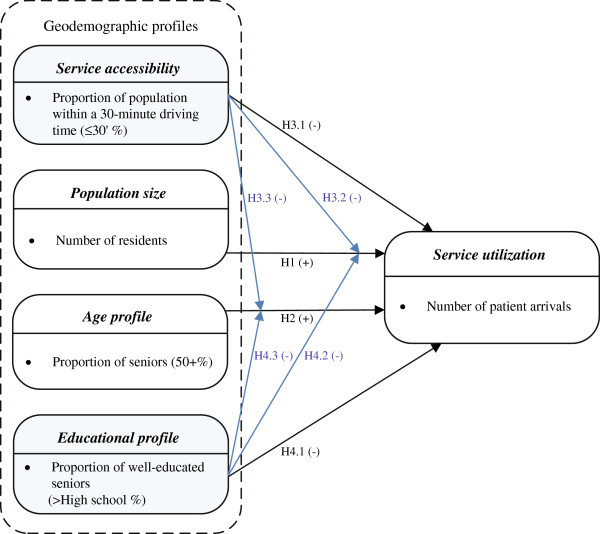
**Research model for this study.** The geodemographic profiles *population size* and *age profile* are positively related to *service utilization*. *Service accessibility* and *educational profile* are negatively related to *service utilization*, and negatively moderate the *population size-service utilization* and *age profile-service utilization* relationships.

The research model, presented in Figure 1, illustrates the hypothesized relationships to be tested in this study.

## Methods

### Data

To test the hypothesized relationships, this study uses secondary data obtained from Statistics Canada and Cardiac Care Network of Ontario from 2004 to 2007. All of the data used in this study are openly available.

**Figure 2:**
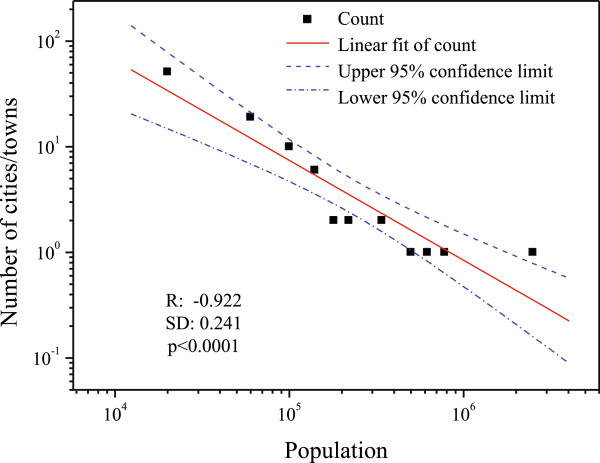
**Population distribution across cities/towns in Ontario.** The city/town population in Ontario follows a power-law distribution (correlation coefficient R=-0.922, standard deviation SD=0.2441, p <0.0001) as shown in this figure. This figure also reveals that our selected cities/towns (with population larger than 40,000) cover a major part (approximately 90.72%) of Ontario’s population.

Geodemographic data with respect to *population size*, *age profile*, and *educational profile* were gathered from Statistics Canada. According to the census data released by Statistics Canada, geodemographic changes in each LHIN were rather gradual every year. For instance, between the 2001 and 2006 Censuses, the population in Ontario grew by approximately 6.6% [22]. Thus, it is reasonable to assume that the 2006 Canadian census [23] would more or less reflect the geodemographics of Ontario over the years between 2004 and 2007. Based on the 2006 Canadian census data [23], we selected 47 major cities/towns in Ontario with population of more than 40,000 to derive the geodemographic profiles for 14 LHINs. The 40,000 population cut-off point was determined such that cities/towns included in our study represented approximately 90.72% of Ontario’s population (as shown in Figure 2). Patients residing in an LHIN may go to other LHINs to receive cardiac surgeries. For instance, 25% of patients residing in the Central West LHIN received treatment from hospitals in the Missisauga Halton LHIN in the fiscal year of 2007/2008 [24]. In view of this, we estimated the population that would potentially utilize the cardiac surgery services in each LHIN, including those residents living in other LHINs, and thereafter derived the corresponding geodemographic profiles.

Specifically, the measurement value for *population size* is calculated by using Equation 1.

(1) $$P_{i}^{\prime }=\overset{14}{\underset{j=1}{\sum }}P_{j}D_{\text{ji}}\;\;(i,j\in [1,14],i\neq j)$$

where *Pi*′ denotes the measurement value of *population size* of LHIN *i*; *P*_*j*_ represents the population size of LHIN *j*; *D*_*ji*_ is the proportion of patients residing in LHIN *j* but receiving services in LHIN *i*. The data representing *D*_*ji*_ were obtained from [24].

The measurement values for *age profile* and *educational profile* for LHIN *i* are calculated by using Equation 2.

(2) $$V_{i}^{\prime }=\frac{\overset{14}{\underset{j=1}{\sum }}V_{j}D_{\text{ji}}}{P_{i}^{\prime }}\;\;(i,j\in [1,14],i\neq j)$$

where *Vi*′ denotes either the proportion of the senior population or the proportion of the well-educated population in LHIN *i*; *V*_*j*_ denotes the number of people aged 50 and above, or the number of well-educated people in LHIN *j*; *D*_*ji*_ is the proportion of patients residing in LHIN *j* but receiving services in LHIN *i*; *Pi*′ is the measurement value of *population size* of LHIN *i*.

In this study, we operationalize *service accessibility* as the proportion of the population residing within a 30-minute driving time to the nearest hospitals providing cardiac surgery services in an LHIN [25]. Here, a 30-minute driving time is selected as a threshold to measure the healthcare service accessibility in accordance with previous work [26,27], and the recommendations from CCN [28]. The driving time from each selected city/town to the nearest hospital that provides cardiac surgery services was estimated by using the “Get directions” function in Google Maps [29]. In Google Maps, a city/town is represented as the center point of its polygonal area [30]. Distinct from a geographical information system (GIS), which estimates driving time based on the lengths of roads and road speed limits [31,32], Google Maps considers the actual traffic conditions on roads. Hence, Google Maps may provide relatively more realistic driving time estimate compared to a GIS. As there may be several routes between a city/town and a hospital in Google Maps, we tabulated the driving time for each selected city/town to all of the hospitals providing cardiac surgery services and selected the route with the shortest driving time to approximate the service accessibility for LHINs. The calculation method for *service accessibility* is shown in Equation 3.

(3) $$SA_{i}=\frac{\sum_{k=1}^{K_{i}}P_{\text{ki}}*\delta_{\text{ki}}}{P_{i}}$$

where *SA*_*i*_ is the service accessibility of LHIN *i*; *P*_*ki*_ is the population size of city/town *k* in LHIN *i*; *K*_*i*_ is the number of cities/towns selected in LHIN *i*; *P*_*i*_ is the population size of LHIN *i*; *δ*_*ki*_ is a parameter to denote whether a city/town *k* in LHIN *i* is within a 30-minute driving time to the nearest hospital.

If the driving time from a city/town *k* in LHIN *i* to its nearest hospital is within 30 minutes, *δ*_*ki*_=1; otherwise, *δ*_*ki*_=0. The geodemographic profiles for the various LHINs are summarized in Table 2.

**Table 2:** The measurement values for geodemographic profiles of LHINs providing cardiac surgery services (2006)

**LHIN ID**	**LHIN name**	***P***^***′***^_***i***_	***A***^***′***^_***i***_ (**%**)	***SA***_***i***_ (**%**)	***E***^***′***^_***i***_ (**%**)
2	South West	762804	32.55	41.05	62.68
3	Waterloo Wellington	671709	29.73	77.69	64.16
4	Hamilton Niagara Haldimand Brant	796559	33.83	51.54	61.25
6	Mississauga Halton	912292	27.54	88.20	71.51
7	Toronto Central	3813418	29.97	100.00	70.12
8	Central	637510	30.07	75.13	69.35
10	South East	198366	33.90	65.10	66.37
11	Champlain	651966	32.80	86.40	74.16
13	North East	189353	37.32	37.27	61.37

*Pi*′: the measurement value for *population size* of LHIN *i*; *Ai*′: the measurement value for *age profile* of LHIN *i*; *SA*_*i*_: the measurement value for *service accessibility* of LHIN *i*; *Ei*′: the measurement value for *educational profile* of LHIN *i*.

Data representing cardiac surgery *service utilization* in 2004-2007 were obtained from the Cardiac Care Network (CCN) of Ontario [33]. As a provincial system that includes 11 hospitals providing cardiac surgery services in Ontario, CCN provides quarterly statistical data on the waiting queue length and the number of completed surgery cases in a month. Based on the CCN data, the average number of cardiac surgery patient arrivals in hospital *i* each month over a quarter *t* ($\text{Arriva}l_{i}^{t}$) can be calculated by adding the number of completed cases to the number of patients waiting in the queue ($\text{NoWai}t_{i}^{t}$), and then subtracting the waiting queue length at time *t*−1 ($\text{NoWai}t_{i}^{t-1}$). An overview of the secondary data on *service utilization* for each hospital examined in this study is shown in Table 3.

**Table 3:** The secondary data about the cardiac surgery service utilization (2004-2007)

**LHIN ID**	**Hospital**	***Service utilization***(**Mean**)
2	London Health Sciences Centre	111
3	St. Mary’s General Hospital	51
4	Hamilton Health Sciences	112
6	Trillium Health Centre	86
7	St. Michael’s Hospital	88
7	Sunnybrook Hospital	71
7	University Health Network	143
8	Southlake Regional Health Centre	64
10	Kingston General Hospital	53
11	University of Ottawa Heart Institute	91
13	Hôspital Régional de Sudbury	38

Note: *Service utilization* is operationalized, or measured, as the number of patient arrivals in a month for a hospital.

### Statistical analysis

The Partial Least Squares (PLS)-based structural equation modeling (SEM) software SmartPLS [34] was employed to test the hypothesized relationships. A powerful second-generation multivariate data analysis technique, SEM is preferable to traditional statistical tools (e.g., regression and ANOVA) in that it is efficient in constructing latent variables that cannot be measured directly, and testing complex relationships among observed and latent variables [12,14]. Moreover, PLS-based SEM, when compared with LISREL (i.e., another major type of SEM), has the advantage of theory development, and thus is more appropriate in exploratory modeling [14]. In this study, all of the latent variables, including *population size*, *age profile*, *service accessibility*, *educational profile*, and *service utilization*, are modeled as reflective constructs (i.e., constructs viewed as causing, as opposed to being caused by, the observed variables) [35].

To test both the direct and the moderating effects hypothesized in this study, we conducted a two-step test as follows: 

•Step 1: testing the direct effects of *population size* and *age profile* on healthcare *service utilization*;

•Step 2: exploring the direct and the moderating effects of *educational profile* and *service accessibility* on *service utilization*.

## Results

The research hypotheses in this study are tested with secondary data on the service utilization of cardiac surgery in Ontario and the relevant geodemographic factors in the years between 2004 and 2007 (16 quarters in total). The mean and standard deviation of all the variables examined in this study are summarized in Table 4.

**Table 4:** Mean and standard deviation of aggregated data

**Variable**	**Mean**	**Standard deviation**	**Min**	**Max**
*Population size*	784907	367484	189353	1271139
*Age profile*				
50+%	31.60	2.63	27.54	37.32
*Service accessibility*				
≤30’ %	67.90	19.59	37.27	100.00
*Educational profile*				
>High school %	67.38	4.24	61.25	74.16
*Service utilization*				
Number of patient arrivals in a month	82	34	16	211

### Measurement model

The common evaluation metrics for model fitting in PLS-based SEM consist of Cronbach’s alpha, construct reliability, and average variance extracted. In this study, as we have utilized one observed variable for each latent variable, the Cronbach’s alpha, construct reliability, and average variance extracted of each latent variable are equal to 1.

### Hypothesized effects of *population size* and *age profile* on *service utilization*

**Figure 3:**
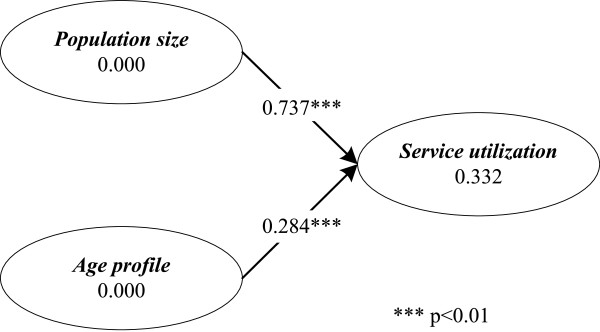
**SEM test results — the effects of*population size* and*age profile* on*service utilization*.** The results of PLS-based SEM testing show that *population size* and *age profile* are both positively related to *service utilization*.

As Figure 3 reveals, in support of **H1** and **H2**, both *population size* and *age profile* have significant positive effects on *service utilization* with path coefficients of *β*=0.737 (*t*=13.205,*p*<0.01) and *β*=0.284 (*t*=5.051,*p*<0.01), respectively. These results lend credence to previous findings that a larger population size [1,15] and a greater proportion of residents older than 50 [17,18] in a geographic area imply more cardiac surgery patients in the hospital(s) of that area.

### Hypothesized effects of *service accessibility* and *educational profile*

**Figure 4:**
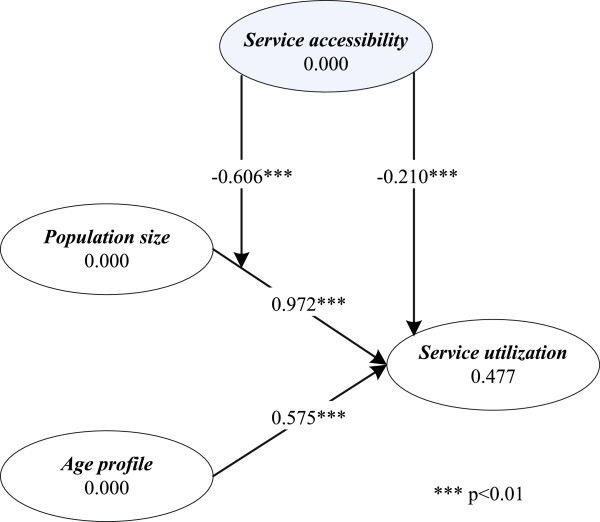
**SEM test results —*service accessibility* as moderator.** The results of PLS-based SEM testing show that *service accessibility* has a direct negative effect on *service utilization*, and a negative moderating effect on the *population size-service utilization* relationship.

As Figure 4 shows, in support of **H3.1** and **H3.2**, *service accessibility* is negatively related to *service utilization* (*β*=−0.210,*t*=2.101,*p*<0.01), and it weakens the effect of *population size* on *service utilization* (*β*=−0.606,*t*=5.240,*p*<0.01). The findings suggest that the more accessible an LHIN is in terms of healthcare services (i.e., with more individuals residing within a 30-minute driving time to the nearest hospital providing cardiac surgery services), the fewer the patient arrivals for any one particular hospital in this LHIN and the weaker the effect of *population size* on *service utilization*. However, hypothesis **H3.3** is not supported by our data (*β*=−0.070,*t*=0.661,*p*>0.05), indicating the absence of the moderating effect of *service accessibility* on the relationship between *age profile* and *service utilization*.

**Figure 5:**
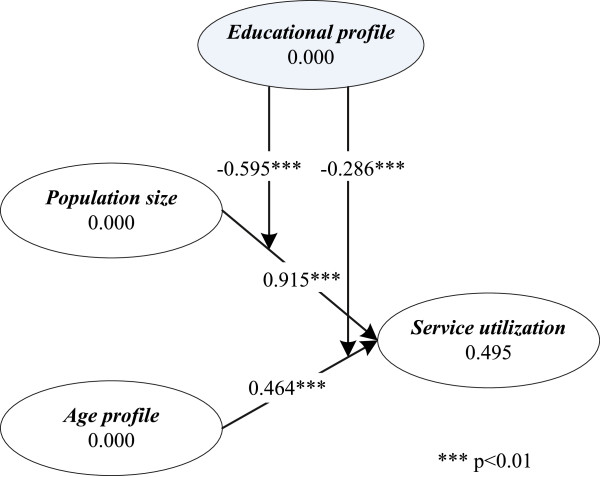
**SEM test results —*educational profile* as moderator.** The results of PLS-based SEM testing show that *educational profile* has a negative moderating effect on the *population size-service utilization* relationship, and the *age profile-service utilization* relationship.

As Figure 5 reveals, **H4.1** is not supported by our data (*β*=0.050,*t*=1.088,*p*>0.1), suggesting the absence of the direct influence of *educational profile* on patient *service utilization* for cardiac surgery. However, in support of **H4.2** and **H4.3**, our results reveal that *educational profile* weakens the effects of *population size* and *age profile* on *service utilization*, with the path coefficients being *β*=−0.595 (*t*=7.592,*p*<0.01) and *β*=−0.286 (*t*=4.987,*p*<0.01), respectively. This suggests that the effects of *population size* and *age profile* on *service utilization* in a well-educated LHIN is not as strong as in a less well-educated LHIN.

Table 5 summarizes the results of hypothesis testing.

**Table 5:** Hypothesis testing results

**Hypotheses**	**Supported?**
H1, H2, H3.1, H3.2, H4.2, H4.3	Fully supported
H3.3, H4.1	Not supported

## Discussion

Meeting the needs of a population is one of the most important considerations when allocating healthcare resources in Canada, and worldwide [9]. Previous research has advocated the allocation of resources according to the needs of the population as assessed by an estimation method [9] that considers demographic-based indicators (e.g., age, education, and smoking) [36,37]. However, examining traditional estimation methods for service needs, Kephart and Asada [37] noted substantial differences between estimated and real needs in some regions. A possible explanation for the biased estimation is that the needs estimation method is simply a linear combination of all of the considered factors, without considering how these factors interact with one another. Therefore, an in-depth understanding of the potential interactions among geodemographic factors (with certain factors moderating the effects of others on healthcare service utilization) can shed light on the design of better estimation methods for healthcare service needs. Further, as LHINs are sub-provincial administrative units responsible for planning and funding healthcare services for their corresponding geographic areas in Ontario [13], our study not only uncovers interesting relationships between LHINs’ geodemographic factors and healthcare service utilization, but also provides valuable knowledge for LHIN administrators to consider in their planning and/or managing of healthcare service resources.

In this paper, we have demonstrated that *service accessibility* has a significant moderating effect on the *population size-service utilization* relationship, and that *educational profile* exerts significant moderating effects on both the *population size-service utilization* relationship and the *age profile-service utilization* relationship, which are novel findings that have not been reported previously. The results of our analysis confirm our prediction that *service accessibility* is negatively associated with *service utilization*, and that it weakens the effect of *population size* on *service utilization*. The results suggest that the more healthcare services are accessible in an area, the fewer cardiac surgery patient arrivals any one particular hospital in that area will have. Take the Hamilton Niagara Haldimand Brant LHIN (LHIN 4) and its neighbor, the Mississauga Halton LHIN (LHIN 6) as examples. In 2007, the proportions of patients receiving cardiac surgery services in their resident LHINs (referred to as the inside-LHIN proportion) were 82% and 72%, respectively [24], whereas the service accessibilities for the two LHINs were approximately 51.54% and 88.20%, respectively, as shown in Table 2. Because both LHIN 4 and LHIN 6 have only one hospital in their own areas, the higher accessibility of LHIN 6 (compared to LHIN 4) suggests that there are more accessible hospitals in the LHINs surrounding LHIN 6 than in those surrounding LHIN 4. As a result, patients dwelling in LHIN 4 are less likely to visit hospitals in other LHINs, compared to those dwelling in LHIN 6, and thus the inside-LHIN proportion for LHIN 4 is higher than that for LHIN 6. Accordingly, we can expect that for LHINs with better accessibility to cardiac surgery services (e.g., LHINs 3, 6, 7, and 11 as shown in Table 2), the pressure of population growth in each of these LHINs on the hospital(s) within the LHIN may decrease.

In contrast, the negative but insignificant moderating effect of *service accessibility* on the relationship between *age profile* and *arrival* may be related to the fact that older people are more willing to visit a familiar hospital or a hospital with familiar physicians [21]. Consequently, service accessibility in an LHIN, which reflects patients’ options in healthcare services, may have little effect on the senior population’s decisions in choosing cardiac surgery services.

The negative moderating effects of *educational profile* suggest that the effect of *population size* and *age profile* on *service utilization* is less pronounced in a well-educated population than it is in a less well-educated population. A possible explanation is that well-educated individuals, including those in old age, may have healthier lifestyles [10]. These individuals also incline to receive routine physical examinations and engage in self-care behavior [11]. Consequently, they are less likely to develop severe cardiovascular disease that requires cardiac surgery services [38]. As illustrated in Table 4, the educational profiles of LHINs varied from 61.25% to 74.16%, with a mean value of 67.38% and standard deviation of 4.24% in 2006. This suggests that the effects of population growth and aging on patient arrivals in each LHIN may vary depending on the educational profiles of the LHIN. Specifically, as shown in Table 2, LHINs 6, 7, 8, and 11, which have more educated populations (indicated by higher-than-average educational profiles), may bear a lighter burden of patient arrivals due to population growth and aging, compared to other LHINs.

In addition, previous research has identified population growth and aging as two important factors driving the need for healthcare services in Ontario [39], and thus affecting patient arrivals. Likewise, our findings reveal a significant relationship between *population size* and *service utilization*, and between *age profile* and *service utilization*. This finding suggests that, for healthcare administrators, monitoring the trends in population growth and aging is an effective precautionary approach to providing sustainable healthcare services.

Finally, prior literature has noted the significant positive effect *service utilization* exerts on hospital wait time, an important performance indicator [40,41]. Our findings suggest that geodemographic factors, such as *population size*, *age profile*, *service accessibility*, and *educational profile*, may indirectly affect wait time for cardiac surgery services via their influence on patient arrivals. Therefore, healthcare administrators should consider the role that geodemographic factors may play in their effort to improve wait time for healthcare services.

### Future research

This study concentrates on investigating the relationships between geodemographic profiles and the utilization of healthcare services at an LHIN level. However, since cities/towns or communities contained in each LHIN may have distinct geodemographic profiles such as age and education, further research may zoom into the LHINs to investigate the effects of geodemographic profiles at a city/town/community level to gain more insights into their impacts. In addition to the geodemographic profiles examined in this study, it would also be desirable to investigate the effects of other factors on healthcare service utilization. For instance, the spatial distribution of risk factors for cardiovascular disease (e.g., co-morbidity of diabetes [42]) may dictate cardiac surgery service utilization.

Moreover, it would be interesting to explore whether our findings still hold for other cardiac care services such as regular checkups, diagnostic cardiac catheterization, and non-invasive percutaneous coronary interventions. This is because cardiac surgery is invasive and commands relatively scarce resources, and thus the geographic accessibility to cardiac surgery is quite limited when compared to other cardiac care services. For example, in 2005, 18 hospitals in Ontario provided angiography tests, while only 11 hospitals performed cardiac surgery services [43]. Further, the purposes and risks for receiving cardiac surgery and for receiving other cardiac care services are also different, thus the service utilization patterns of cardiac surgery may differ from those of other cardiac care services.

Future research may also be carried out to extend our SEM testing method. In this study, the moderating effects of *educational profile* and *service accessibility* on the *population size-service utilization* relationship and the *age profile-service utilization* relationship are tested separately due to the collinearity between the two moderators. In the future, the moderating effects of the two factors may be tested simultaneously in a comprehensive model by using primary data. Moreover, in this study, each latent variable has only one indicator. Future research may examine additional indicators for each of the latent variables so as to capture more dimensions of these variables.

## Conclusions

This study extends previous research by exploring the moderating effects of geodemographic factors on healthcare service utilization, in addition to examining the direct effects of such geodemographic factors. Unlike previous research, this study investigates the hypothesized relationships by employing an SEM data analysis technique and utilizing secondary data on cardiac surgery service utilization in Ontario, Canada. The results of this study reveal that geodemographic changes due to population growth and aging may significantly affect cardiac surgery service utilization. Moreover, geographic accessibility to healthcare services and educational profile exert significant effects on patient arrivals for cardiac surgery, both as direct antecedents and as moderators. Our findings demonstrate the importance of considering the geodemographic profiles of a geographic area, and sometimes its neighboring areas, when allocating healthcare service resources, thus strategically improving service utilization and reducing wait time.

## Competing interests

The authors declare that they have no competing interests.

## Authors’ contributions

LT, JL, and BX conceived and designed the experiments. LT performed the experiments and drafted the manuscript. LT, JL, and BX revised the manuscript. All of the authors read and approved the final manuscript.

## Pre-publication history

The pre-publication history for this paper can be accessed here:

http://www.biomedcentral.com/1472-6963/13/239/prepub
